# Early Molecular Insights into Thanatin Analogues Binding to *A. baumannii* LptA

**DOI:** 10.3390/molecules28114335

**Published:** 2023-05-25

**Authors:** Kathryn K. Oi, Kerstin Moehle, Matthias Schuster, Oliver Zerbe

**Affiliations:** Department of Chemistry, University of Zurich, Winterthurerstrasse 190, CH-8057 Zurich, Switzerland

**Keywords:** Gram-negative bacteria, antimicrobial resistance, *A. baumannii*, LPS transport, LptA, thanatin, thanatin analogues, NMR structure, binding constants

## Abstract

The cationic antimicrobial ß-hairpin, thanatin, was recently developed into drug-like analogues active against carbapenem-resistant Enterobacteriaceae (CRE). The analogues represent new antibiotics with a novel mode of action targeting LptA in the periplasm and disrupting LPS transport. The compounds lose antimicrobial efficacy when the sequence identity to *E. coli* LptA falls below 70%. We wanted to test the thanatin analogues against LptA of a phylogenetic distant organism and investigate the molecular determinants of inactivity. *Acinetobacter baumannii* (*A. baumannii*) is a critical Gram-negative pathogen that has gained increasing attention for its multi-drug resistance and hospital burden. *A. baumannii* LptA shares 28% sequence identity with *E. coli* LptA and displays an intrinsic resistance to thanatin and thanatin analogues (MIC values > 32 µg/mL) through a mechanism not yet described. We investigated the inactivity further and discovered that these CRE-optimized derivatives can bind to LptA of *A. baumannii* in vitro, despite the high MIC values. Herein, we present a high-resolution structure of *A. baumannii* LptAm in complex with a thanatin derivative **7** and binding affinities of selected thanatin derivatives. Together, these data offer structural insights into why thanatin derivatives are inactive against *A. baumannii* LptA, despite binding events in vitro.

## 1. Introduction

*Acinetobacter baumannii* (*A. baumannii*) is an opportunistic Gram-negative nosocomial pathogen that has gained increasing notoriety for its multi-drug resistance (MDR). In a systematic review, *A. baumannii* harboring one or more drug resistance mechanisms was attributed to 132,000 deaths in 2019 [1]. The most prominent drug resistances were against carbapenem, fluoroquinolones, anti-pseudomonal, aminoglycosides, and cephalosporins [1]. The World Health Organization (WHO) has identified carbapenem-resistant *A. baumannii* as a critical pathogen for which new antibiotics are urgently needed [2].

Gram-negative bacteria are difficult to penetrate with antibiotics due to the double bi-layer membrane capsule coated by lipopolysaccharide (LPS) [3]. Conventional antibiotics have intracellular targets that inhibit essential pathways: cell wall biosynthesis (e.g., β-lactams, glycopeptides), protein synthesis (e.g., aminoglycosides, tetracyclines, phenicols), DNA synthesis (e.g., fluoroquinolones, trimethoprim), and RNA synthesis (e.g., rifamycins) [4]. Small molecular drugs (<600 Da) may achieve membrane passage by simple diffusion through the lipid bilayer or diffusion through outer membrane porins [5]. In contrast, alternative antibiotics that often exceed 600 Da and/or carry a positive charge, such as the cationic antimicrobial peptide (CAMP) polymyxin-type (colistin) and aminoglycosides, achieve cell passage by transiently perturbing LPS at the outer membrane (OM) via the so-called self-promoted uptake pathway [6,7] to enter and reach their targets, LPS at the inner membrane (IM) and intracellular targets, respectively [8,9,10,11,12]. 

LPS is the hallmark antigen that constitutes the OM of Gram-negative bacteria. It is a large amphipathic molecule (usually 10–20 kDa) containing a lipid anchor, Lipid A, and an extensive glycan structure comprising a constant core domain and a variable O-antigen domain; the latter can comprise hundreds of carbohydrates. LPS serves as a selective barrier to foreign agents in the extracellular environment, and protects the structural integrity and rigidity of the cell membrane [11]. Transportation of LPS to the extracellular surface of the OM is facilitated by seven essential LPS transport proteins, LptA-G, that form a molecular bridge spanning the periplasm [13]. The periplasmic domains of LptC, LptA and LptD share high structural homology and are composed of ß-strands that fold into ß-jellyrolls. Therein, hydrophobic residues face inward and hydrophilic residues face into the periplasmic environment. Shuttling across the periplasm from the IM to the OM was proposed to proceed in a PEZ-like fashion, such that the amphipathic LPS molecules orient with the lipid A moiety into the hydrophobic core of the protein bridge and the O-antigen into the periplasm [14]. 

The OM of *A baumannii* is coated with lipooligosaccharide (LOS), a variant of LPS without the O-antigen, as well as other capsular polysaccharides such as poly-ß-1,6-N-acetylglucosamine (PNAG) and glycoproteins that confer drug- and desiccative-resistance [15]. When faced with a selective pressure such as colistin, *A. baumannii* mutant clones have remarkable adaptability, and the ability to remodel their OM by modifying the drug target or inactivating LOS biosynthesis altogether. LPS/LOS biosynthesis and transport genes are essential in most Gram-negative bacteria such as *E. coli* and *K. pneumoniae,* and thus remain susceptible to colistin. However, recent studies have demonstrated that LOS is not essential in *A. baumannii,* and that colistin treatment can select *A. baumannii* LOS-deficient mutant clones from cultivated and clinical strains [16]. 

In the emerging colistin-resistant *A. baumannii* strains, differential expression of genes that modify the Lipid A target have been observed. Modification of the Lipid A phosphate groups to phosphoethanolamine (PEtN) or galactosamine (GalN) removes the negative charges which likely weaken colistin binding. More specifically, the upregulation or mutation of the two-component system *pmrAB* regulating these PEtN and GalN modifications has been implicated to confer colistin resistance [17,18]. Additionally, in the LOS biosynthesis pathway, the constitutive expression of *lpxM*, an acyltransferase, modifies Lipid A with hepta-acylation, and has also been linked to colistin resistance [19]. In cases of viable *A. baumannii* with total loss of LOS, the *lpxACD* genes are commonly mutated and the LOS biosynthesis pathway inactivated [16]. Without LOS, the capsular polysaccharide PNAG and lipoproteins are upregulated, which seems to keep the structural integrity of the OM intact [20]. The colistin-resistant *A. baumannii* clones come with a fitness cost, and increase sensitivity to other antibiotics in combination therapy or to host immune cells [16,21,22]. 

In the wake of MDR *A. baumannii* strains disseminating, innovation for new antibiotics targeting this critical pathogen is urgent. The OM components of *A. baumannii* remain an interesting landscape for new antimicrobial targets. Recently, LptA of the LPS transport system was identified as a new druggable target in Enterobacteriaceae for the naturally occurring cationic antimicrobial peptide, thanatin [23], and its more potent drug-like derivative peptides [24]. These compounds were shown to exert bactericidal effects in Enterobacteriaceae by competitively binding to the N-terminus of LptA and disrupting LPS transport [24]. Interestingly, *A. baumannii* is phenotypically resistant to thanatin and its derivatives (MIC values > 32 µg/mL), and its LPS transport system remains poorly characterized. We sought to investigate the inactivity at the structural and biophysical levels that could explain its resistance. Herein, we describe the interaction of *A. baumannii* LptA with previously described CRE-optimized thanatin analogues. We present a high-resolution structure of the LptAm-analogue **7** complex as well as the binding affinities of protein–peptide systems. Together, these data offer structural insights into why thanatin derivatives are inactive against *A. baumannii* LptA, despite observing binding events in vitro. 

## 2. Results

In our recent work, we demonstrated that thanatin analogues are potent antibiotic contenders against carbapenem-resistant *E. coli*, *K. pneumoniae* and a panel of other Enterobacteriaceae [24]. The lead compound **7** reported favorable properties such as high affinity for LptA and potent antimicrobial activity against Enterobacteriaceae in vitro and in vivo. *A. baumannii* LptA has a low sequence identity to that of *E. coli* (28%), and the former has no sensitivity to the peptides (MIC > 32 µg/mL) (Table 1). Despite its intrinsic resistance profile, thanatin and thanatin analogues were tested with *A. baumannii* LptA, a phylogenetically distant homolog to the parent target (*E. coli*), with unexpected insights. We followed the format of [24] by first solving the structure of a recombinant monomeric LptA (LptAm) in complex with **7**, followed by determination of binding constants for **7** and some selected predecessors of **7**: compounds **1**, **2**, **5** and **6** (Table 2). Details on the peptides in this paper have been previously described in [24]. 

### 2.1. Protein Characterization and Quality Control 

**Figure 1:**
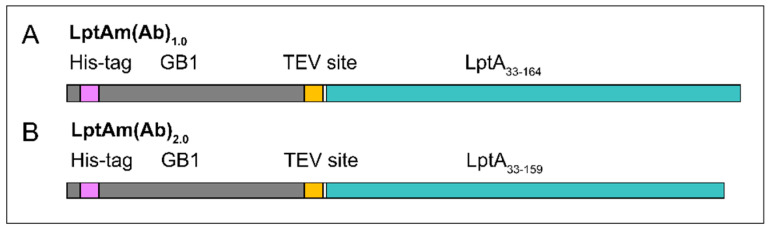
*A. baumannii* protein constructs. The LptAm(Ab) protein constructs (green) were recombinantly expressed with a fusion protein GB1 (grey) containing a histidine tag (purple) and a TEV cleavage site (yellow). The final products contain a glycine artefact (G32, white) not present in the wild-type protein sequence. LptAm(Ab)_1.0_ (**A**) spans from L33 to R164 and LptAm(Ab)_2.0_ (**B**), L33-G159.

Thanatin and thanatin analogues bind to Enterobacteriaceae LptA at the N-terminus [23,24]. Our protein of interest, LptA, is a soluble periplasmic protein that has been shown to homooligomerize in vitro, and full-length LptA of Enterobacteriaceae resulted in aggregation and precipitation. Previous work reported that a C-terminal truncation of the last ß-strand resulted in a well-behaving monomeric *E. coli* LptA (LptAm) in solution [25]. Thus, we conceived a similarly truncated *A. baumannii* LptA construct. The *E. coli* LptAm protein sequence functioned as a homology model, and sequence alignment guided the C-terminal truncation for the *A. baumannii* homolog. The initial construct, *A. baumannii* LptAm_1.0_ (LptAm(Ab)_1.0_) spans from L33 to R164, beginning after the signal peptide (1–32) and omitting the last ß-strand (165–181) (Figure 1A).

**Figure 2:**
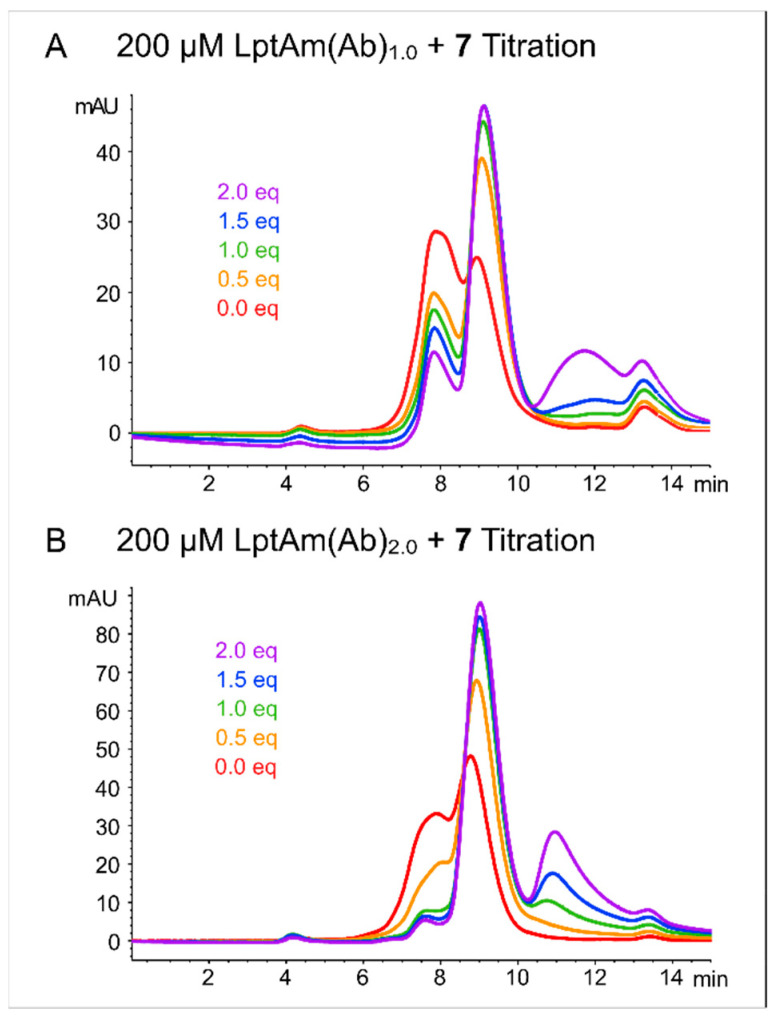
Analytical SEC chromatograms of the LptAm constructs in the absence and presence of **7**. LptAm(Ab)_1.0_ (**A**) and LptAm(Ab)_2.0_ (**B**) were prepared and measured in the same way, with protein concentrations of 200 µM and the same equivalents of **7** in the titration.

The characterization of LptAm(Ab)_1.0_ in solution showed heterogeneity of protein states, especially in absence of the ligand, as seen in the absorption traces at 280 nm in an analytical SEC chromatogram (Figure 2A). Upon increasing equivalents of analogue **7**, ligand binding was indicated by a smaller hydrodynamic radius and the increased peak intensity for the bound monomeric protein (retention time of 8–10 min). The secondary species (retention time of 7–8 min) reduced, but could not be completely resolved, even with two-fold excess concentrations of ligand. Excess ligand was observed to elute between 10 and 13 min. An additional peak (retention time between 13–14 min) was observed in all titration samples, corresponding to residual imidazole from the elution buffer; SDS-PAGE analysis of the purified protein fraction showed no indications of contamination or degradation (Figure S1). LptAm(Ab)_1.0_ was used for our NMR analysis, resulting in high-quality spectra. 

While small amounts of aggregated species do not cause significant problems in the NMR analysis, the unresolved shoulder species of LptAm(Ab)_1.0_, corresponding to the peak at a retention time of 7–8 min, presented a problem for binding affinity assays. Herein, strict stoichiometries and known concentrations of monomeric protein in solution are required. Thus, we sought to design a new protein construct with improved stability of the monomer in solution. LptAm(Ab)_2.0_ is the same as LptAm(Ab)_1.0_, except for a further 5-residue truncation at the C-terminus, spanning from L33 to G159 (Figure 1B). Inspection of LptAm(Ab)_2.0_ using analytical SEC measured at 280 nm, suggesting that the apo state behaves similarly to LptAm(Ab)_1.0_ in solution. However, upon the addition of the ligand in the same step-wise titration as before, the heterogeneity of LptAm(Ab)_2.0_ is dramatically reduced, and the absorbance intensity of the monomeric species is two-fold higher than that of LptAm(Ab)_1.0_ (Figure 2B). The SEC experiments in Figure 2 were prepared with the same protein concentration of 200 µM, and ligand increments from 0.0 to 2.0 equivalents. LptAm(Ab)_2.0_ exhibited improved properties in solution, especially in the presence of the ligand, and thus was used for all binding assays. 

All *A. baumannii* protein constructs were recombinantly cloned downstream of the soluble protein GB1, and heterologously expressed in *E. coli* BL21(DE3) cells, as described in the Supplementary Materials.

### 2.2. Structural Studies Using Nuclear Magnetic Resonance (NMR)

The apo state of LptAm(Ab)_1.0_ was unstable, as indicated by poor-quality NMR spectra and precipitation within the first 24 h at room temperature. The addition of **7** to LptAm(Ab)_1.0_ stabilized the protein to remain folded throughout the NMR measurements, and the structure of thanatin analogue **7** binding to LptAm(Ab)_1.0_ was elucidated. Uniformly ^13^C, ^15^N-labeled protein in complex with **7** was used in a standard set of 3D triple-resonance experiments for backbone assignments. The structure of bound-LptAm(Ab)_1.0_ was derived using 3D nuclear Overhauser effect spectroscopy (NOESY) spectra. **7** contains many non-canonical amino acids, and could not be recombinantly produced for isotope filtered NOESY experiments. Instead, perdeuteration of LptAm(Ab)_1.0_ and simple homonuclear experiments were executed for peptide assignments in the complex. Proton signals of the free peptide were assigned in 2D homonuclear NOESY, COSY and TOCSY spectra and were used to guide sidechain assignments. Intermolecular NOEs were distinguished from intermolecular ones with a ^13^C-filtered NOESY. From our set of spectra, 92% of backbone resonances, 84% of sidechain resonances, and 74% of aromatic resonances were assigned in the well-defined regions of the structure. We achieved a high-resolution structure of the complex with an average RMSD of the backbone and heavy atom residues of 0.66 ± 0.12 Å and 1.02 ± 0.08 Å, respectively, excluding the unstructured loops and termini regions (Table S5). The accession codes generated for the PDB and BMRB databases are 8ONU and 34,802, respectively.

**Figure 3:**
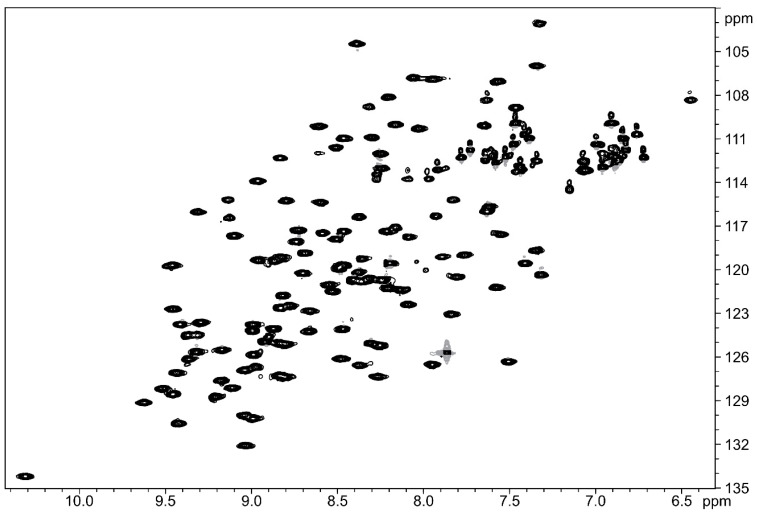
[^15^N,^1^H]-HSQC of 300 µM LptAm(Ab)_1.0_ with 2.4 equivalents of **7** measured at 600 MHz, 298 K.

The high MIC values of the thanatin peptides suggested little to no antimicrobial activity against *A. baumannii*, so we were surprised to observe high-quality NMR spectra of the complex LptAm(Ab)_1.0_-**7**. The [^15^N,^1^H]-HSQC spectrum of ^15^N-labeled LptAm(Ab)_1.0_ with 2.4 equivalents of **7** shows good signal dispersion, indicating that LptAm(Ab)_1.0_ is well folded and stable in the presence of the peptide (Figure 3). Assignments of the ^15^N backbone resonances are shown in Figure S2.

**Figure 4:**
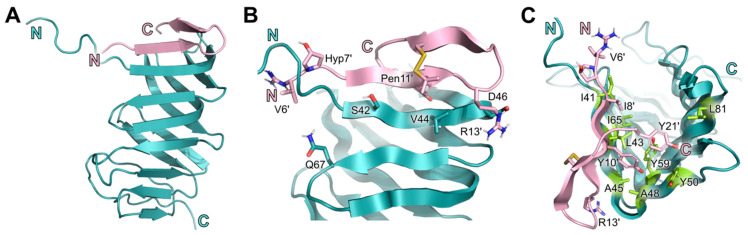
Structure of the complex of **7** binding to LptAm(Ab)_1.0_. The elucidated structure of LptAm(Ab)_1.0_-**7** as seen from the side (**A**), depicting only the backbone and secondary structures. LptAm(Ab)_1.0_ is composed of ß-strands with a tertiary ß-jellyroll fold, and the loops and termini are unstructured. Important sidechains point outwards into the periplasm (**B**) and within the hydrophobic core (**C**). (**A**) is depicted as flat sheets, whereas (**B**,**C**) are depicted as fancy sheets in PyMOL. The sidechains in (**C**) are depicted in yellow to emphasize their orientation. N and C notations represent the N-terminus (peptide in pink, protein in green) and C-terminus (peptide in pink, protein in green) of their respective protein chains. Additional depictions of the interface can be found in Figure S4.

Similar to the homologs of Enterobacteriaceae, LptAm(Ab)_1.0_ comprises of ß-strands which fold into a ß-jellyroll, stabilized by the hydrophobic network of inward-facing sidechains (Figure 4A,B). Resonances from the ^15^N NOESY indicated that the N-terminus has a helical turn (D36, R37, N38) that is more flexible than the corresponding helical turns observed in the *E. coli* and *K. pneumoniae* LptAm structures. The C-terminus (residues 150–164) was not well assigned in our spectra, and is likely unfolded, with its resonances located in the random coil region. General dihedral angle restraints were derived from chemical shifts using TALOS+, and used to predict the secondary structure LptAm(Ab)_1.0_ (Figure S3) [26]. 

**7** docks to LptAm(Ab)_1.0_ residues I41, L43, V44, A45, and A48 of the first ß strand in a parallel orientation, as indicated by the intermolecular NOEs observed: backbone–backbone (e.g., L43 HN–Y10′ HA), backbone–side chain (e.g., I41 HN–I8′ HD1), and sidechain–sidechain (e.g., V44 HG–Pen 11′ HG) (Figure 4B,C and Figure S4). Nonsequential contacts with spatially nearby residues Q67 and L81 were also observed at the complex’s interface (e.g., Q67 HE22–VAL6′ HG and L81 HD–Y21′ HD) (Figure S4C). A comprehensive list of intermolecular NOEs is reported in Table S6. 

Importantly, the extensive network of hydrophobic residues in the ß-jellyroll core observed in *E. coli* and *K. pneumoniae* LptAms is markedly reduced in LptAm(Ab)_1.0_. Notably, I8′ of **7** stacks efficiently against a hydrophobic patch consisting of I41, L43 and I65 (Figure S5). Two important binding determinants of LptAm for ligand docking observed in *E. coli* and *K. pneumoniae* are L45 and F54 in the hydrophobic core, and in *A. baumannii*, both residues are replaced by tyrosine, Y50 and Y59, respectively. The changes to tyrosine likely lead to clashes with Y10′ and Y21′ of **7**, and suitable pi–pi stacking interactions cannot be achieved (Figure 4C and Figure S4). This could explain the observed lower affinity and the reduced number of intermolecular NOEs (Figure S8).

### 2.3. Fluorescence Polarization Binding Studies 

As previously mentioned, a second *A. baumannii* LptA protein construct (LptAm(Ab)_2.0_), truncated at G159 of the C-terminus had improved monomeric behavior in solution and was used for binding affinity assays. The protein underwent routine checks for purity via a Coomassie-stained SDS-PAGE (Figure S1), and its binding to **7** in a titration series was observed using analytical SEC (Figure 2B), before proceeding with fluorescence polarization (FP). The fluorescent label, Alexa647, was conjugated to thanatin at the first position, as described in [24]. In brief, G1′ of thanatin is replaced with an L-lysine derivative and undergoes a copper catalyzed reaction with Alexa647 to produce the fluorescently labeled peptide, **than-FL**, which was used as the reporter in all FP assays. 

**Figure 5:**
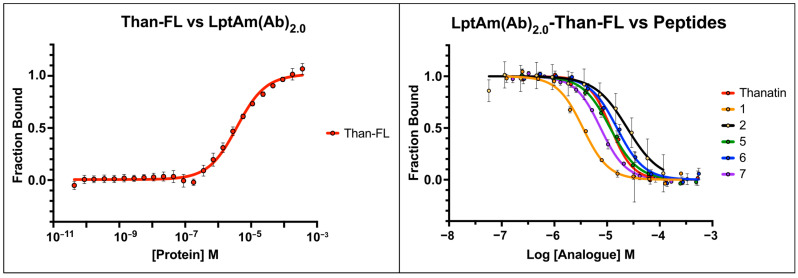
Fluorescence polarization binding assays for thanatin and thanatin analogues. Fluorescently labeled thanatin (**than-FL**) was measured against LptAm(Ab)_2.0_ in a direct binding assay (**left**). IC_50_ curves are shown for thanatin and analogues in competition assays (**right**).

The dissociation constant (K_D_) of **than-FL** was first determined in a direct binding assay and measured as 3.7 ± 0.5 µM (Figure 5, left) (Table 3). Subsequently, competitive binding assays were performed to determine the inhibition constants (K_I_s) of the unlabeled thanatin derivatives **1**, **2**, **5**, **6**, and **7**. For each thanatin derivative tested, a serial dilution was prepared in triplicate on a single 96-well plate, to which a 1:1 mixture of LptAm(Ab)_2.0_ and **than-FL** was added. The displacement of **than-FL** restores fluorescence depolarization, and the resulting curve reveals the IC_50_ for each unlabeled peptide (Figure 5, right). Determination of the K_I_s was achieved from the empirically derived IC_50_ values and K_D_ of **than-FL** using the Cheng–Prusoff equation [27]. Native thanatin displaced its fluorescent cognate **than-FL** with a K_I_ of 3.2 ± 0.4 µM (Table 3). Thanatin derivatives **2**, **5**, and **6** were equivalent or weaker binders, with K_I_s ranging from 3.1–6.5 µM, whereas **1** and **7** had increased affinities relative to thanatin, with K_I_s of 0.9 ± 0.1 µM and 2.0 ± 0.1 µM, respectively (Table 3). 

### 2.4. Examining the Affinities of Thanatin Derivatives 

Thanatin, the naturally occurring 21-mer, binds to LptAm(Ab)_2.0_ with a K_I_ of 3.2 ± 0.4 µM, approximately the median between all the peptides tested. The current lead peptide against CRE, **7**, had an improved binding of 2.0 ± 0.1 µM. Without the guanidine moiety, peptide **6**, doubles the K_I_ to 4.1 ± 0.4 µM. Peptide **5**, another 16-mer similar to **6** and **7**, was an interesting peptide for Enterobacteriaceae, as it displayed the highest affinity against the thanatin-resistant Q62L-LptA mutant. However, its affinity with LptAm(Ab)_2.0_ is comparable to that of thanatin, and warranted less interest in the context of *A. baumannii*. Peptide **1**, a 19-mer, displayed the highest affinity to LptAm(Ab)_2.0_. Interestingly, **1** was also a strong contender for *E. coli* (MIC 0.063 µg/mL and K_I_ 1.1 nM) but showed less favorable ADMET properties [24]. We speculate the higher affinity for LptAm(Ab)_2.0_ could be attributed to the norleucine (Nle) at position 21′. Nle has a linear aliphatic four-carbon long sidechain, which could improve the hydrophobic packing into the protein’s hydrophobic core and to L81, explaining the higher affinity. The aliphatic sidechain likely packs more efficiently against the LptA interface, mainly residues Y50 and Y59, than the methionine of thanatin and the tyrosine of **7**.

### 2.5. Conserved Binding Mode of 7 to LptAm across Phyla

**Figure 6:**
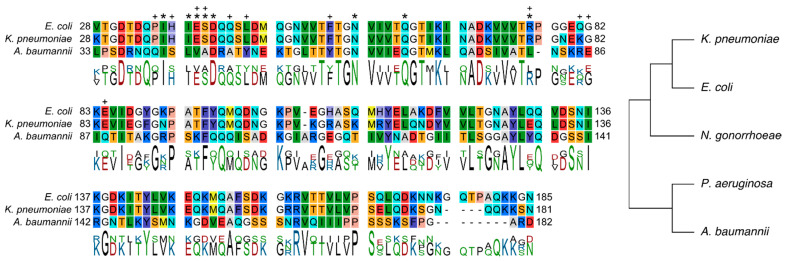
Conservation in LptA sequence of three WHO priority 1 Gram-negative pathogens. The sequence alignment of LptA in Gram-negative pathogens, *E. coli*, *K. pneumoniae*, *A. baumannii*. The asterisks (*) above I36, I38, E39, S40, D41, N57, Q62 and R76 of *E. coli* LptAm indicate common residues involved in the binding of analogue **7** by amino acid and/or the spatial position across all three species (**left**). Of note, the single point mutation Q62L in Enterobacteriaceae LptA was the dominant, recurring mutation conferring thanatin resistance in *E. coli* and *K. pneumoniae*. The crosses (+) above P35, H37, E39, L45, F54, R76, and E84 of *E. coli* LptAm as well as R85 and E86 of LptAm(Ab) indicate notable 7 binding determinants for Enterobacteriaceae that are different or not observed in LptAm(Ab). The numbers adjacent to the sequences indicate residue number in their respective protein sequences. The LptA phylogenetic tree for a selection of Gram-negative pathogens, including the three discussed in this paper, emphasizes the distance between *A. baumannii* and *E. coli* (**right**).

In our previous paper, we examined thanatin derivatives interacting with two closely related LptAs of the Enterobacteriaceae family. The LptA primary structures of *E. coli* and *K. pneumoniae* are largely conserved in sequence (88%), structure, and binding mode of thanatin derivatives [24]. On the other hand, the sequence identity of *E. coli* LptA to *A. baummannii* is only 28% (Table 1). *A. baumannii* belongs to the Moraxellaceae family related to Enterobacteriaceae only via a common phylum Pseudomonadota. The sequence alignment of the LptAm protein constructs demonstrates that there is some conservation across phylogenetically distant Gram-negative bacteria not only in the primary and tertiary structures of LptA, but also in their binding mode to **7** (Figure S9). The first ß-strand (I38–Q43) of *E. coli* LptAm where **7** docks is generally conserved in *A. baumannii* as well, the first ß-strand corresponding to residues I41–A48 and validated by intermolecular pairings at conserved positions (Figure 6). For example, position Q62 in the Enterobacteriaceae aligns with Q67 of *A. baumannii* and is part of a conserved motif of Q-G-T across all three species (Figure 6). It has been noted in our previous work that Q62 stabilizes the observed N-terminal helical turn and improves the stability of the ß-jellyroll. For *E. coli* and *A. baumannii*, the glutamine at positions 62 and 67, respectively is close to V6′ (4.0–5.0 Å) and plays a role at the interface. 

In Enterobacteriaceae, three intermolecular electrostatic interactions were observed as important binding determinants of **7** to LptAm, and validated by molecular dynamics (MD) simulations. R13′ of **7** docks to LptAm(Ab)_1.0_ in a nearly identical mode as reported in *E. coli* and *K. pneumoniae*. Through MD simulations, the guanidine group of R13′ was observed to interact electrostatically with the electronegative groups of E39, D41 and N57 of Enterobacteriaceae LptAms [24]. In *A. baumannii*, R13′ of **7** docks into a similar pocket of LptAm(Ab)_1.0_ (V44, D46, N62) with intermolecular NOEs observed between V44 and R13′ (Figure S6). V44 is positioned at the conserved site E39 of LptAm in Enterobacteriaceae, and sufficiently close (3.4–4.7 Å) to R13′ to postulate similar contacts for these four residues (R13′, V44, D46 and N62). D46 and N62 of LptAm(Ab)_1.0_ are conserved in alignment with D41 and N57 of Enterobacteriaceae LptAms. Another notable position is R76 of LptA in Enterobacteriaceae, in which the guanidine group of R76 forms an intermolecular salt bridge with the carboxyl group of Y21′ that helps to anchor **7** and stabilize the binding state of the complex. In LptAm(Ab)_1.0_-**7**, a similar interaction is observed for R85 and the Y21′, but is notably more flexible and not present in all conformations of the NMR bundle (Figure S6). MD simulations of this intermolecular interaction verify its flexibility and only transient electrostatic interactions (Figure S7). It is also noted that L81 of LptAm(Ab)_1.0_ aligns with the R76 position in Enterobacteriaceae LptAm, and that the sidechain of L81 is sufficiently close (4.0 Å) to Y21′ to form aliphatic–aromatic interactions; however, these interactions are only weak, and significant flexibility of Y21′ is observed. Lastly, electrostatic interactions of E84 in *E. coli* LptAm with Gua’ of **7** were significant in the binding mode. In *A. baumannii*, the position is substituted with a Q88 and makes no contributions to **7** binding (Figure 6). Interestingly, E86 in LptAm(Ab)_1.0_ is oriented towards the Gua’ of **7** in a minor subset of the NMR conformations, inferring some electrostatic dynamics, albeit orders of magnitude weaker than what we can observe in the Enterobacteriaceae complexes (Figure S6). Additionally, the MD simulation suggested no relevant intermolecular interactions between E86 in LptAm(Ab)_1.0_ and **7** due to marked flexibility. 

There were also notable binding determinants observed in the Enterobacteriaceae complexes that were not detected in LptAm(Ab)_1.0_-**7**. As mentioned previously, L45 and F54 of Enterobacteriaceae LptA were not only significant for peptide binding, but also for extending the hydrophobic network of the LptAm core and adding stability to the overall protein fold. In *A. baumannii*, the corresponding positions are tyrosines, Y50 and Y59, which effectively reduce the hydrophobic core to a hydrophobic patch and sterically clash with the tyrosines Y10′ and Y21′ of the peptide (Figure S4). Another critical intermolecular interaction observed in *E. coli* LptAm-**7** is the stacking of P35 of the protein with Hyp7′ of **7,** which strengthens the docking of the peptide to the protein. Analogous to this position in LptAm(Ab)_1.0_ is Q40, and no intermolecular NOEs were observed. H37 of *E. coli* LptAm was also involved at the interface, whereas in *A. baumannii*, no intermolecular NOEs were detected at its corresponding residue S42. Additionally, the substantial decrease in observed NOEs between protein residues and Y21′ also indirectly indicates its flexibility in the complex, especially when comparing it to Enterobacteriaceae LptAms (n = 3 NOEs for *A. baumannii* vs. 16 for *E. coli* and 18 for *K. pneumoniae*) (Figure S8). Overall, the higher binding affinity of **7** to Enterobacteriaceae LptAm can be explained by the greater number of protein residues involved in the binding of **7** at the interface, accompanied by a higher total number of intermolecular NOEs (Figure S8). *A. baumannii* has fewer protein–peptide interactions and greater flexibility at the interface, explaining its reduced affinity although intermolecular interactions of peptide residues V6′, I8′, Y10′, Pen11′, R13′, and Y21′ were observed across all the species studied (Figure S8).

Because **7** was optimized against Enterobacteriaceae and shows no antimicrobial activity against *A. baumannii*, it was surprising for us to observe still a low micromolar binding affinity and a binding mode similar to *E. coli* and *K. pneumoniae* for the *A. baumannii* system. Despite their distance in phyla and low sequence identity, conservation of LptAm structure and function are apparent (Figure S9). While the micromolar affinity of **7** to LptAm of the Moraxellaceae family is not sufficient for therapeutic use, it uncovers key binding determinants of the protein and justifies the inactivity of the thanatin peptides at the molecular level. We further speculate that these conserved sites of LptA in *A. baumannii* are not only important for binding thanatin analogues, but are also likely to be key binding determinants for building the Lpt bridge across the periplasm.

## 3. Discussion and Outlook 

Thanatin and thanatin derivatives are cationic antimicrobial peptides (CAMPs) that have been shown to target the LPS transport protein, LptA, in the periplasm of Enterobacteriaceae [24]. However, the potency of the described thanatin derivatives loses efficacy after the sequence identity to *E. coli* LptA falls below 70% [24]. *A. baumannii* LptA has a sequence identity of 28%, and its resistant phenotype to thanatin and thanatin derivatives (MICs > 64 µg/mL) corroborates the reported threshold. Deeper investigation into the inactivity of thanatin peptides against *A. baumannii* revealed that the peptides can bind to LptAm(Ab)_1.0_ in vitro, and molecular determinants specific to the *A. baumannii* LptA identified could explain this. 

Our elucidation of the *A. baumannii* LptAm in complex with the thanatin derivative **7** revealed the surprising likeness of LptAs to Enterobacteriaceae, despite the phylogenetic distance. The tertiary structure of *A. baumannii* LptAm assumes the same ß-jellyroll fold, including the characteristic N-terminal helical turn. Moreover, the binding mode of **7** to LptAm(Ab)_1.0_ has features that were observed for the LptAs of *E. coli* and *K. pneumoniae*. **7** binds in a parallel orientation to the first ß-strand of LptAm, and includes the intermolecular interactions at conserved binding positions Q67 and L81 in *A. baumannii* to Q62 and R76 in *E. coli* (Figure 6). Interestingly, Q62 was also important for stabilizing the fold by preserving the N-terminal helical turn, and the loss of this turn, as seen in the single mutation Q62L-LptA, conferred resistance to the native compound thanatin in Enterobacteriaceae [24]. While the N-terminal helical turn was also observed in LptAm(Ab)_1.0_, its role in stabilizing the overall protein fold was not indicated. Additionally conserved electrostatic interactions could also be interpreted for some conformations of the LptAm(Ab)_1.0_-**7** NMR bundle, but these need further validation. 

The differences in the *A. baumannii* LptA primary structure also justify why the binding affinities are micromolar, and three orders weaker than Enterobacteriaceae LptAs. The thanatin derivatives bind to Enterobacteriaceae LptAs with low nanomolar affinity in part due to efficient packing of the peptidyl Y10′ and Y21′ residues against the LptA hydrophobic residues L45 and F54. In *A. baumannii*, the latter residues at the N-terminal interface are replaced by Y50 and Y59. We suspect that the hydrophobic core becomes overcrowded by introducing these sterically more demanding sidechains. Consequently, crucial interactions to build the binding interface are missing, resulting in a less stable protein–peptide complex. 

Presently, we do not know exactly which mechanism is driving thanatin-resistance in *A. baumannii*, whether the reduced antimicrobial activity is simply related to poor permeation of the cell envelope [28] and/or that the peptides have insufficient binding affinity to LptA of *A. baumannii*. Our in vitro results suggest that the low affinity of the peptides to the *A. baumannii* LptA (in comparison to *E. coli*) could explain the lack of antimicrobial activity; however, other resistance factors in vivo cannot be ruled out. Little is known about how thanatin and its derivatives enter the *Enterobacteriaceae* cells to reach their periplasmic target. LptD has been described as another interacting partner of thanatin [23,29], and could be a possible entry point. Alternatively, electrostatic interactions between the cationic charges of the peptide and the negative charges on LPS would suggest cell entry via the self-promoted uptake pathway [7,30,31]. A study by Ma and colleagues corroborates that thanatin binds to LPS with low micromolar affinity, displacing the endogenous divalent calcium ions and permeating the OM of *E. coli* [32]. If electrostatic interactions between LPS and thanatin peptides are necessary for cell entry, then the strain-dependent LPS displayed on the OM—or complete lack thereof—could determine whether thanatin and thanatin analogues are active or inactive against Gram-negative pathogens in vivo. 

In *Enterobacteriaceae*, LPS and its biosynthesis and transport proteins are effective targets, as LPS is essential for cell viability. While this essentiality holds true for most Gram-negative organisms, *A. baumannii* is an exception. In *A. baumannii*, the Lipid A target can be modified with PEtN, or GalN [18,33], or mutant clones can inactivate Lipid A biosynthesis and survive as LPS-deficient [16], rendering it not only colistin-resistant but possibly thanatin-resistant as well. However, further investigations are needed and are beyond the scope of our early findings.

We have provided a developing profile of thanatin derivatives binding to *A. baumannii* LptA despite its lack of antimicrobial activity (MIC values > 32 µg/mL). Our structural insights help to explain why *A. baumannii* LptA is not a target of thanatin antibiotics thus far. Future investigations into the *A. baumannii* Lpt bridge and protein–protein interactions, specifically LptA-LptA or LptA-LptC, would advance our understanding of thanatin analogues in the *A. baumannii* system. *A. baumannii* is an opportunistic critical pathogen armed with a diverse set of resistance genes, and continues to outcompete our antibiotics. The more we investigate its pathogen-specific AMR pathways, the more effectively new drugs can be designed. 

## 4. Materials and Methods

All methods were performed as described in [24,34], but are briefly paraphrased here. 

### 4.1. Constructs, Cloning, and Protein Expression

The C-terminally truncated monomeric LptA (LptAm) constructs were designed from a homology model aligned with Enterobacteriaceae LptAm sequences, as described in [23,24]. The constructs were cloned from *A. baumannii* ATCC 17978 genomic DNA using respective primers along the *lptA* sequence. The truncated *lptA* inserts were cloned downstream of a fusion protein, protein G B1, domain derived from Streptococcus *spp*. of the vector pEM, as described in [35]. Plasmids were replicated in *E. coli* DH5α competent cells in LB medium, and isolated using miniprep kits (QIAGEN Aarhus, Denmark). Transformed *E. coli* BL21(DE3) cultures were grown in LB medium, and in minimal 9 (M9) medium when experiments required isotopically labeled nuclei (e.g., U-^2^H, U-^13^C, U-^15^N). Cells were induced using 0.5–1.0 mM IPTG at OD_600_ 0.6–1.0 and grown overnight at 25 °C with moderate shaking.

### 4.2. Protein Purification

Upon harvesting, cells were spun down at 5000 RPM at 4 °C for 15 min and resuspended in lysis buffer (50 mM NaPi pH 7, 150 mM NaCl, 20 mM imidazole, 10% glycerol). Cells in the lysis buffer were supplemented with 2 mg/mL lysozyme (Roth, Karlsruhe, Germany), 0.1 mg/mL DNase (Roche, Basel, Switzerland), 5 mM MgCl_2_, 1 mM PMSF (Roth, Karlsruhe, Germany), and then subjected to sonication (Branson Digital Sonifier 250, Emerson Electric Co., St. Louis, MO, USA) over ice in two rounds of 5 min with 30% output, 1 s on and 3 s off. Lysate was centrifuged at 18,000 RPM at 4 °C for 30 min. Soluble lysate was extracted and subjected to 0.22 µm filtration before loading on a nickel column. Protein was purified in a two-step NiNTA purification process with an overnight TEV cleavage incubation before collecting the untagged protein of interest in the reverse column flow through fraction. Purified protein was buffer exchanged to SEC buffer (50 mM NaPi pH 7, 150 mM NaCl) with an overnight dialysis at 4 °C. The purity of protein was checked using analytical size exclusion chromatography (SEC) and SDS-PAGE stained with Coomassie Blue (Figure S1). 

### 4.3. NMR Spectroscopy, Assignments, and Structure Calculations

#### 4.3.1. Free Peptide Assignment

Analogue **7** was dissolved in acetate buffer pH 4.0 and assigned from 2D homonuclear NOESY, COSY and TOCSY spectra. The structure of the free peptide was calculated from the 300 ms NOESY spectrum.

#### 4.3.2. Protein–Peptide Complex Assignment and Structure Calculation

A standard set of 3D triple-resonance experiments using uniformly ^13^C, ^15^N (U-^13^C, U-^15^N)-labeled protein in complex with analogue **7** was used [36]. Intra-ligand NOEs were unambiguously assigned by ^2^H, ^15^N isotopically labeling LptAm, in which the residual proton density was less than 1%; 2D [^1^H,^1^H], ^15^N-filtered NOESY was recorded upon addition of analogue **7** [37]. Intermolecular sidechain NOEs were acquired using a ^13^C-edited, ^13^C-filtered NOESY experiment. Upper distance restraints were derived from 80 ms ^15^N-resolved 3D NOESY spectrum, and ^13^C-resolved 3D NOESY spectra centered on aliphatic (39 ppm) and aromatic (120 ppm) carbons. NOESY spectra were iteratively and automatically assigned using the CYANA macro *noeassign* [38]. Torsion angle restraints were derived from backbone chemical shifts using the program TALOS+ [26]. The solution structure complex *A. baumannii* LptAm(Ab)_1.0_-**7** was deposited to the PDB (BMRB) databases under accession codes 8ONU (34802).

### 4.4. Fluorescence Polarization (FP) Assays 

All binding affinity assays were performed in triplicate on Optiplate-96F microplates (Perkin Elmer, Schwerzenbach, Switzerland) and executed at ambient temperature (25 °C). Protein stocks were purified and concentrated in SEC buffer (50 mM NaPi pH 7, 150 mM NaCl), and if necessary diluted to the desired concentration using FP buffer (50 mM NaPi pH 7, 150 mM NaCl, 0.05% Tween20). Peptide stocks and fluorescently labeled peptide stocks were made in FP buffer. A 1:1 serial dilution of 24 wells was prepared first by adding 100 µL of the unlabeled protein (e.g., LptAm(Ab)_2.0_) or peptide (e.g., **1**, **2**, **5–7**), starting at high concentrations, 100–500 µM, in well one. Then, 50 µL was taken from the first well and added to 50 µL of FP buffer in the subsequent well, homogenized and repeated for the remaining of the 24-well series. Then, 50 µL of 2x stock of the fluorescently labeled thanatin (**than-FL**) was added to the dilution series. For the direct binding assays, 10 µM than-FL was used as the 2x stock, and for the indirect binding assays, a 1:1 mixture of 20 µM Lpt protein variant and 20 µM than-FL was used as the 2x stock; the final working concentrations of the **than-FL** were 5 µM and 10 µM, respectively. Plates were left to incubate overnight at 4 °C before fluorescence polarization (FP) measurements. Anisotropy was measured using the Tecan Safire^2^ plate reader using a 10 nm bandwidth and setting the excitation and emission wavelengths to 635 nm and 670 nm, respectively. The G-factor (G) was also determined empirically as 1.094 for the instrument and included in all the anisotropy calculations.

For direct binding assays, fluorescence anisotropy (*r*) was calculated using the parallel and perpendicular polarized intensities using the equation $r=\frac{I_{∥}-I_{⊥}*G}{I_{∥}+2I_{⊥}*G}$. Anisotropy for a blank, the fluorescently labeled peptide only (**than-FL** only), was measured and subtracted from r to yield *r*’. The *r*’ data were normalized and fit to the One site—Total model on GraphPad Prism 9. A nonspecific binding factor (NS) was determined using a linear regression model (*y* = NS × *X* + b) at the highest concentrations and incorporated into the fit. The dissociation constants (*K_D_*s) were calculated using the total protein concentrations, using the equation $y=\frac{B_{max}X}{K_{D}+X}$ [39,40].

For the indirect binding assays, *r* was calculated in the same manner as the direct binding assays. To control for non-specific binding between **than-FL** and competitors, **than-FL** alone was added to a replicate dilution series of the unlabeled peptide on the same plate. The control was subtracted from *r* to yield *r*’. Data were fit to a sigmoidal interpolation model on GraphPad Prism 9, and an IC_50_ was generated for each unlabeled peptide or protein measured. The inhibition constants (*K_i_*s) were calculated using the Cheng–Prusoff equation, $K_{i}=IC_{50}\times {\left(1+\frac{L_{T}}{K_{D}}\right)}^{-1}$, in which the total concentration of **than-FL** (*L_T_*) was used as well as the *K_D_* previously derived from the peptide’s cognate direct binding assay. 

### 4.5. Protein Sequence Analysis

LptA gene and protein sequences of interest were extracted from public domain databases: uniprot, KEGG and NIH BLAST. The sequence alignment was performed using CLC Main Workbench Analysis 7.8.1 (QIAGEN, Aarhus, Denmark), the phylogenetic tree (Maximum likelihood statistical analysis, nearest-neighbor interchange method) was generated using Molecular Evolutionary Genetics Analysis (MEGA 11.0.8), and the final figure was compiled in Affinity Designer (1.10.4). Sequence identity (%) was calculated using BLAST (blastp suite-2 sequences).

## Figures and Tables

**Table 1:** LptA sequence identity to *E. coli* and MIC values (µg/mL).

Strain	*E. coli* ATCC 25922	*K. pneumoniae* ATCC 43816	*P. aeruginosa* ATCC 27853	*A. baumannii* DSM 30008
LptA Identity	100%	88%	30%	28%
Thanatin	1	2	>64	>64
**1**	0.063	0.250	>64	>32
**2**	0.031	0.125	>32	>32
**5**	0.063	0.250	>32	>32
**6**	0.125	0.250	>32	>32
**7**	0.063	0.125	>64	>64

**Table 2:** Sequences of thanatin and thanatin derivatives.

	Position																				
Peptide	1′	2′	3′	4′	5′	6′	7′	8′	9′	10′	11′	12′	13′	14′	15′	16′	17′	18′	19′	20′	21′
Thanatin	G	S	K	K	P	V	P	I	I	Y	C	N	R	R	T	G	K	C	Q	R	M
**1**			K	K	P	V	P	I	I	Y	C	N	R	R	T	G	K	C	Dab	R	Nle
**2**			K	K	P	V	P	I	I	Y	Pen	N	R	Dab	T	DDab	K	C	Dab	R	Y
**5**						V	Hyp	I	I	Y	Pen	N	R	Dab	T	DDab	K	C	Dab	Dab	Y
**6**						V	Hyp	I	T	Y	Pen	N	R	Dab	T	DDab	K	C	Dab	R	Y
**7**					Gua	V	Hyp	I	T	Y	Pen	N	R	Dab	T	DDab	K	C	Dab	R	Y

Note: Gua, guanidine moiety; Hyp, hydroxyproline; Pen, penicillamine; Dab, diaminobutyric acid; Nle, norleucine. Apostrophes are added to the peptide residues to distinguish them from protein residues.

**Table 3:** Summary of binding affinities between thanatin peptides and LptAm(Ab)_2.0_.

**Protein**	**Ligand**	**Than-FL Conc. (µM)**		**K_D_ ± 95CI (µM)**	**Experiment**
LptAm(Ab)_2.0_	**Than-FL**	5		3.7 ± 0.5	Direct
**Protein-FL**	**Ligand**	**Than-FL conc. (µM)**	**IC_50_ ± 95CI (µM)**	**K_I_ ± 95CI (µM)**	**Experiment**
LptAm(Ab)_2.0_-than-FL	Thanatin	10	11.8 ± 1.5	3.2 ± 0.4	Competition
LptAm(Ab)_2.0_-than-FL	**1**	10	3.5 ± 0.3	0.9 ± 0.1	Competition
LptAm(Ab)_2.0_-than-FL	**2**	10	24.1 ± 6.3	6.5 ± 1.7	Competition
LptAm(Ab)_2.0_-than-FL	**5**	10	11.5 ± 0.7	3.1 ± 0.2	Competition
LptAm(Ab)_2.0_-than-FL	**6**	10	15.4 ± 1.3	4.1 ± 0.4	Competition
LptAm(Ab)_2.0_-than-FL	**7**	10	7.5 ± 0.4	2.0 ± 0.1	Competition

Note: Total concentration of reporter, **than-FL**, are reported for each assay.

## Data Availability

Not applicable.

## Supplementary Materials

The following supporting information can be downloaded at: https://www.mdpi.com/article/10.3390/molecules28114335/s1; Table S1: Nucleotide sequences of primers and plasmid constructs; Table S2: Sequences of protein constructs as expressed and after two-step purification; Table S3: Composition of Media and Buffers; Table S4: LptA sequences of selected pathogens, excluding the signal peptide, used in the sequence alignment and phylogenetic tree; Table S5: Statistics from the NMR structure calculations; Table S6: List of all intermolecular NOEs (by type) of complex *A. baumannii* LptAm-**7**; Figure S1: Coomassie-stained SDS PAGE of Protein Expression and Purification for LptAm(Ab)_1.0_ (left) and LptAm(Ab)_2.0_ (right); Figure S2: Annotated protein-observed [^15^N,^1^H]-HSQC of ^15^N-labeled LptAm(Ab)_1.0_ in complex with analogue **7**; Figure S3: TALOS+ Prediction of Secondary Structure; Figure S4: Binding mode of LptAm(Ab)_1.0_-**7**; Figure S5: The intermolecular hydrophobic patch of LptAm(Ab)_1.0_-**7** depicted as spheres; Figure S6: Postulated electrostatics between LptAm(Ab)_1.0_ and **7**; Figure S7: Minimum distances between the guanidine group of R85 of LptAm(Ab)_1.0_ (Arg85GUA) and the carboxyl group of Y21′ of 7 (TYR21_COO) as a function of time during the MD simulations of complex LptAm(Ab)_1.0_-**7**; Figure S8: Intermolecular NOEs between **7** and LptAm of *A. baumannii* (black), *E. coli* (green), and *K. pneumoniae* (blue); Figure S9: Comparing the binding mode of analogue **7** to LptAs of different Gram-negative pathogens.

## Sample Availability

Requests for peptide samples should be directed to Spexis AG. Plasmids for protein constructs are available upon request from the authors.
