# Supplementary material for: Early Molecular Insights into Thanatin Analogues Binding to A. baumannii LptA
Source: Molecules. 2023 May 25;28(11):4335. doi: 10.3390/molecules28114335 (PMC10254193; doi:10.3390/molecules28114335)
Supplement: Supplementary file 1 [file molecules-28-04335-s001.zip › molecules-2407928-supplementary.pdf]

Supplementary Materials

Early Molecular Insights into Thanatin Analogues Binding to *A. baumannii* LptA

Kathryn K. Oi, Kerstin Moehle, Matthias Schuster and Oliver Zerbe *

Department of Chemistry, University of Zurich, Winterthurerstrasse 190, CH-8057 Zurich, Switzerland

* Correspondence: oliver.zerbe@chem.uzh.ch

Table S1. Nucleotide sequences of primers and plasmid constructs

Construct Name	DNA Sequence (5’-3’)
$LptAm(Ab)_{1.0}$ forward primer	TTTGCTCTTCGCAGGGATTGCCGTCTGACCGTAATCAAC
$LptAm(Ab)_{1.0}$ reverse primer	TTTGGATCCTTATTAACGGTTTGAAGAAGAACCTTGAGC
$LptAm(Ab)_{2.0}$ reverse primer	TTTGGATCCTTATTAACCTTGAGCTTCAACATCACC
6His-GB1-TEV-$LptAm(Ab)_{1.0}$	ATGTCTGGTTCTCATCATCATCATCATCATAGCAGCGGCATCGAAGGC<br>CGCGGCCGCCAGTACAAACTGATCCTGAACGGTAAAACCCTGAAAGG<br>TGAAACCACCACCGAAGCTGTTGACGCTGCTACCGCGGAAAAAGTTTT<br>CAAACAGTACGCTAACGACAACGGTGTTGACGGTGAATGGACCTACG<br>ACGACGCTACCAAAACCTTCACCGTTACCGAAAGCAGCGGCGAAAAC<br>CTGTACTTCCAGGGATTGCCGTCTGACCGTAATCAACAAATTTCGTTA<br>GTGGCAGACCGAGCAACTTATAATGAAAAAACCGGTTTGACGACTTAT<br>ACGGGTAATGTCGTGATTGAGCAGGGCACCATGAAGCTTCAGGCTGA<br>CTCAATTGTGGCTACGCTAAACTCTAAACGTGAAATTCAAACGATCAC<br>TGCTAAAGGTAGACCGTCTAAGTTTCAGCAACAAATAAGTGCTGATAA<br>AGGTATTGCACGCGGTGAAGGACAAACGATTGTTTATAATGCAGATAC<br>AGGTATTATTACCTTGTCTGGCGGTGCATATTTATACCAAGATGGTTCA<br>AGTATTCGCGGTAACACCCTGAAATATAGTATGAATAAGGGTGATGTT<br>GAAGCTCAAGGTTCTTCTTCAAACCGTTAATAA
6His-GB1-TEV-$LptAm(Ab)_{2.0}$	ATGTCTGGTTCTCATCATCATCATCATCATAGCAGCGGCATCGAAGGC<br>CGCGGCCGCCAGTACAAACTGATCCTGAACGGTAAAACCCTGAAAGG<br>TGAAACCACCACCGAAGCTGTTGACGCTGCTACCGCGGAAAAAGTTTT<br>CAAACAGTACGCTAACGACAACGGTGTTGACGGTGAATGGACCTACG<br>ACGACGCTACCAAAACCTTCACCGTTACCGAAAGCAGCGGCGAAAAC<br>CTGTACTTCCAGGGATTGCCGTCTGACCGTAATCAACAAATTTCGTTA<br>GTGGCAGACCGAGCAACTTATAATGAAAAAACCGGTTTGACGACTTAT<br>ACGGGTAATGTCGTGATTGAGCAGGGCACCATGAAGCTTCAGGCTGA<br>CTCAATTGTGGCTACGCTAAACTCTAAACGTGAAATTCAAACGATCAC<br>TGCTAAAGGTAGACCGTCTAAGTTTCAGCAACAAATAAGTGCTGATAA<br>AGGTATTGCACGCGGTGAAGGACAAACGATTGTTTATAATGCAGATAC<br>AGGTATTATTACCTTGTCTGGCGGTGCATATTTATACCAAGATGGTTCA<br>AGTATTCGCGGTAACACCCTGAAATATAGTATGAATAAGGGTGATGTT<br>GAAGCTCAAGGTTAATAA

Table S2. Sequences of protein constructs as expressed and after two-step purification

Protein	Protein Sequence
6His-GB1-TEV-LptAm(Ab)$_{1.0}$	MSGS**HHHHHH**SSGIEGRGRQ**YKLILNGKTLKGETTTEAVDAAT**<br>**AEKVFKQYANDNGVDGEWTYDDATKTFTVTE**SSG**ENLYFQ**GL<br>PSDRNQQISLVADRATYNEKTGLTTYTGNVVIEQGTMKLQADSIV<br>ATLNSKREIQTITAKGRPSKFQQQISADKGIARGEGQTIVYNADTG<br>IITLSGGAYLYQDGSSIRGNTLKYSMNKGDVEAQGSSSNR
LptAm(Ab)$_{1.0}$	**G**LPSDRNQQISLVADRATYNEKTGLTTYTGNVVIEQGTMKLQAD<br>SIVATLNSKREIQTITAKGRPSKFQQQISADKGIARGEGQTIVYNAD<br>TGIITLSGGAYLYQDGSSIRGNTLKYSMNKGDVEAQGSSSNR
6His-GB1-TEV-LptAm(Ab)$_{2.0}$	MSGS**HHHHHH**SSGIEGRGRQ**YKLILNGKTLKGETTTEAVDAAT**<br>**AEKVFKQYANDNGVDGEWTYDDATKTFTVT**ESSG**ENLYFQ**GL<br>PSDRNQQISLVADRATYNEKTGLTTYTGNVVIEQGTMKLQADSIV<br>ATLNSKREIQTITAKGRPSKFQQQISADKGIARGEGQTIVYNADTG<br>IITLSGGAYLYQDGSSIRGNTLKYSMNKGDVEAQG
LptAm(Ab)$_{2.0}$	**G**LPSDRNQQISLVADRATYNEKTGLTTYTGNVVIEQGTMKLQAD<br>SIVATLNSKREIQTITAKGRPSKFQQQISADKGIARGEGQTIVYNAD<br>TGIITLSGGAYLYQDGSSIRGNTLKYSMNKGDVEAQG

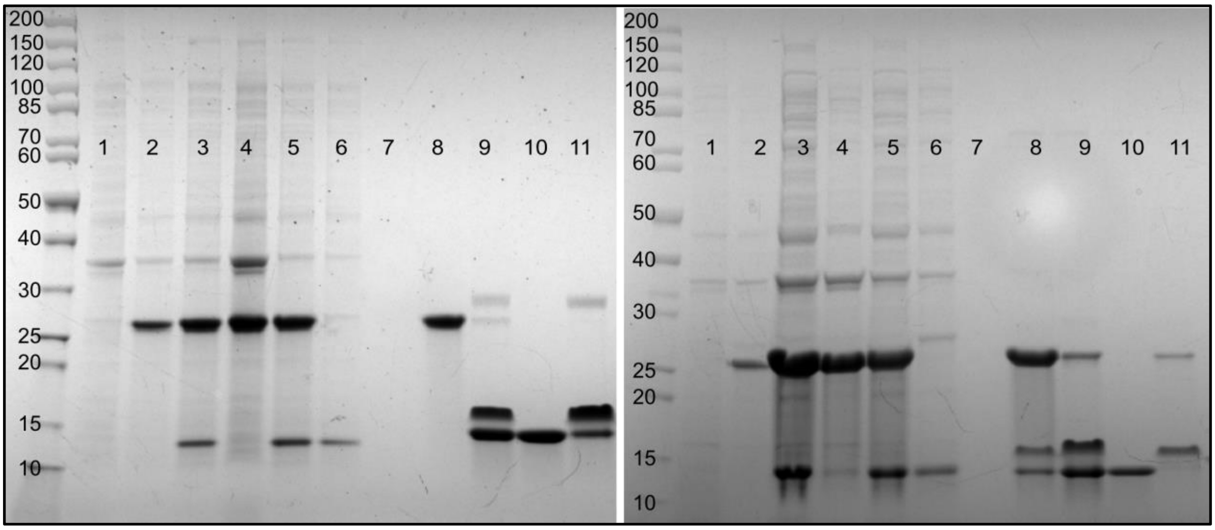

Figure S1. Coomassie-stained SDS PAGE of Protein Expression and Purification for LptAm(Ab)$_{1.0}$ (left) and LptAm(Ab)$_{2.0}$ (right). Protein purification lanes: (1) uninduced expression culture (2) induced expression culture (3) whole cell extract (4) insoluble (pellet) fraction (5) soluble fraction (6) flow through (7) wash (8) GB1-LptAm(Ab) fusion proteins (9) TEV-digested GB1 and LptAm(Ab) (10) Pure LptAm(Ab) (11) His-tagged TEV and GB1 proteins eluted with 500 mM imidazole.

Table S3. Composition of Media and Buffers

Medium	Buffer
Minimum 9 (M9) Medium per 1 L	Na_2HPO_4 (3.0 g), KH_2PO_4 (7.5 g), NaCl (0.5 g), MgSO4 (2mM), Trace Metals (1 mL 1000x stock), Carbenicillin (0.1 g/L), ^{13}C Glucose (3.0 g), NH_4Cl (1.0 g)
Lysogeny Broth (LB) Medium per 1 L	Bacto yeast extract (5.0 g), bacto tryptone (10.0 g), NaCl (5.0 g), Autoclaved
Lysis Buffer	50 mM NaPi pH7, 150 mM NaCl, 20 mM imidazole, 10 % glycerol
Elution Buffer	50 mM NaPi pH7, 150 mM NaCl, 500 mM imidazole, 10 % glycerol
SEC Buffer	50 mM NaPi pH7, 150 mM NaCl
Fluorescence Polarization (FP) Buffer	50 mM NaPi pH7, 150 mM NaCl, 0.05 % Tween20

Table S4. LptA sequences of selected pathogens, excluding the signal peptide, used in the sequence alignment and phylogenetic tree

Organism	LptA protein sequence
P. aeruginosa	LPSDREQPIRVQADSAELDDKQGVAVYRGDVVVTQGSTKLTGNTVTLKQDRNGDIEVVTSVGKPAYYEQKPAPDKDVTKAYGLTIQYFVTQNRVVLIDQAKVIQEGNTFEGEKIVYDTQRQIVNAGRATGSQVTSPRPRIDMVIQPKKKAQ
A. baumannii	LPSDRNQQISLVADRATYNEKTGLTTYTGNVVIEQGTMKLQADSIVATLNSKREIQTITAKGRPSKFQQQISADKGIARGEGQTIVYNADTGIITLSGGAYLYQDGSSIRGNTLKYSMNKGDVEAQGSSSNRVQIIIPPSSSKSFPGARD
N. gonorrhoeae	LQSDSRRPIQIEADQGSLDQANQSTTFSGNVIIRQGTLNISASRVNVTRGGKGGESVRAEGSPVRFSQTLDGGKGTVRGQANNVTYSSAGSTVVLTGNAKVQRGGDVAEGAVITYNTKTEVYTINGSTKSGAKSASKTGRVSVVIQPSSTQKTE
K. pneumoniae	KTGDTDQPIHIESDQQSLDMQGNVVTFTGNVVVTQGTIKINADKVVVTRPGNEKGKEVIEGFGNPATFYQMQDNGKPVKGRASKMRYELQNDYVVLTGNAYLEQLDSNIKGDKITYLVKEQKMQAFSDKGRRVTTVLVPSELQDKSGNQQKKSN
E. coli	VTGDTDQPIHIESDQQSLDMQGNVVTFTGNVIVTQGTIKINADKVVVTRPGGEQGKEVIDGYGKPATFYQMQDNGKPVEGHASQMHYELAKDFVVLTGNAYLQQVDSNIKGDKITYLVKEQKMQAFSDKGKRVTTVLVPSQLQDKNNKGQTPAQKKGN

Table S5: Statistics from the NMR structure calculations

	LptAm(Ab)$_{1.0}$-**7**
**PDB (BMRB) Accession Code**	8ONU (34802)
**NOE distance restraints**	
Total	1280
Intra-residue, i-j=0	327
Sequential, i-j=1	387
Medium-range, 1<i-j<5	125
Long-range, i-j ≥ 5	441
Intermolecular	41
Torsion angle constraints	166
**Structure statistics (20 conformers)**	
CYANA target function value (Å^2)	4.88 ± 0.28
**Satisfaction of Experimental Constraints**	
Distance constraint violation	
Number >0.2 A	13 ± 3
Maximum (**Å**)	0.66 ± 0.09
Torsion angle constraint violations	
Number >5°	0
Maximum (deg)	1.99 ± 0.45
PROCHECK Ramachandran plot analysis	
Residues in favored regions (%)	73.8
Residues in additional allowed regions (%)	24.8
Residues in generously allowed regions (%)	0.9
Residues in disallowed regions (%)	0.5
**RMSD to the average coordinates (Å)**	
Backbone atoms (residues 33-150, 6-21)	1.92 ± 0.51
Heavy atoms (residues 33-150, 6-21)	2.21 ± 0.47
Backbone atoms (regular secondary structure)[a]	0.66 ± 0.12
Heavy atoms (regular secondary structure)[a]	1.02 ± 0.08

[a] protein residues: 42-51,56-82,87-103,108-121,125-137,139-150 analogue **7**: 9’-11’

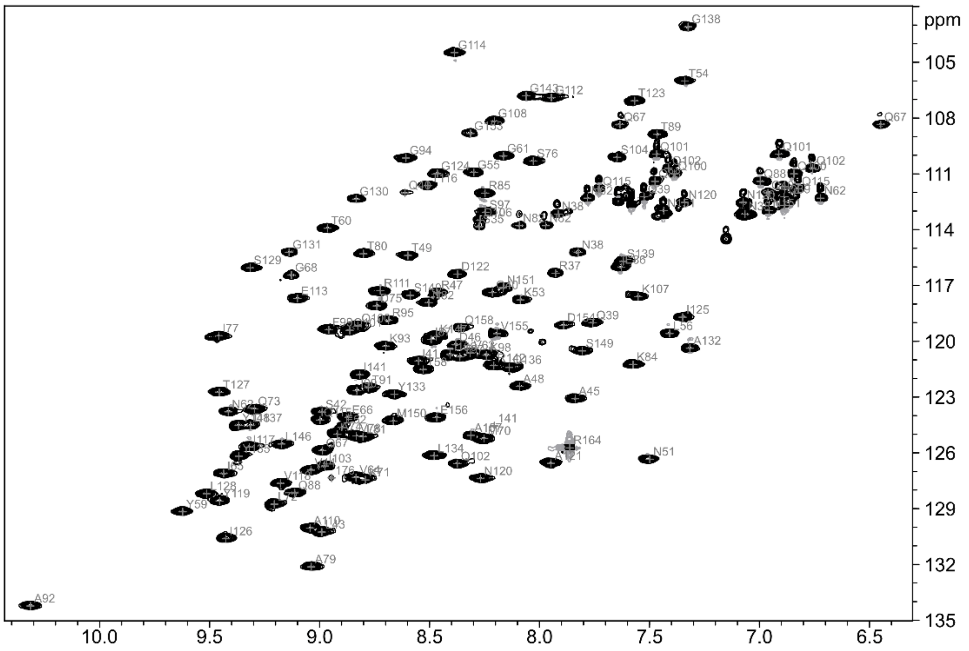

Figure S2. Annotated protein-observed [^{15}N,^{1}H]-HSQC of ^{15}N-labeled LptAm(Ab)$_{1.0}$ in complex with Analogue **7**. The ^{15}N and ^{1}H of the backbone and sidechain amides are annotated. The resonances show good signal dispersion indicated that LptAm(Ab)$_{1.0}$ is well folded in presence of **7**.

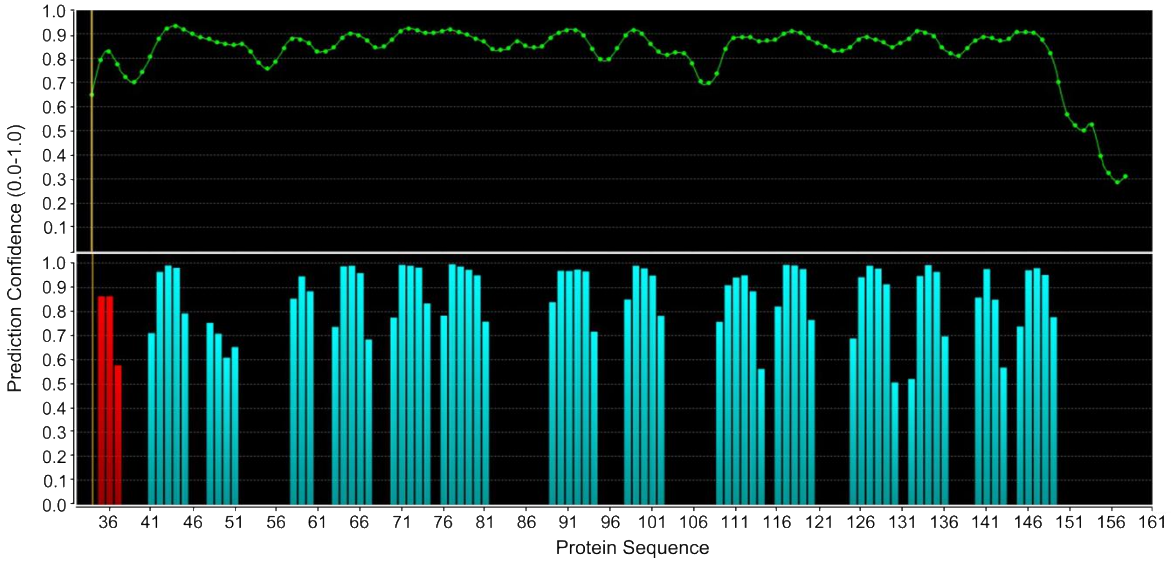

Figure S3. TALOS+ Prediction of Secondary Structure. Output graphs were adapted from TALOS+ as described in (*1*). Primary structure and NMR chemical shift assignments are used in the online software to predict the random coil index (RCI) S2 value (top) and the secondary structure (bottom). Colors for the secondary structure are encoded as: red for ∝-helix, cyan for ß-strand, and areas of no prediction would suggest a flexible (unstructured) region (e.g. terminus or loop). The y-axis is in confidence ranging from 0 to 1 and the x-axis represents the residue numbers of the protein sequence.

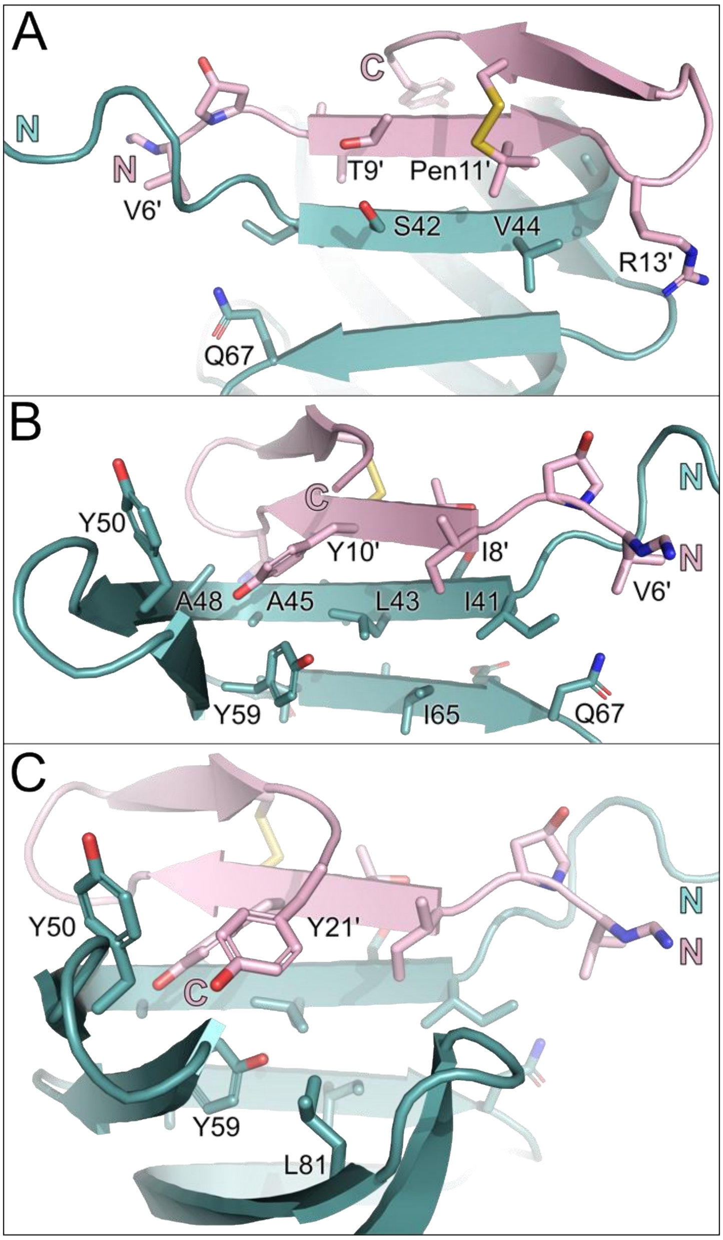

Figure S4. Binding mode of LptAm(Ab)$_{1.0}$-**7**. A closer look at the interface of the protein-peptide complex is shown. **7** binds to LptAm in a parallel orientation in which we see outward-facing and exposed residues V44 and Q67 interact with Pen11' and R13', and V6' respectively (**A**). Looking inside the protein core, a network of aliphatic residues I41, L43, A45, A48 interact with I8' and Y10'. The aforementioned hydrophobic patch I8', I41, L43 and I65 is annotated as well as the bulky sidechains of Y50 and Y59 for reference; the sidechain of Y21' is not shown for simplicity (**B**). The C-terminal Y21' is proximal to L81 located in the third loop of the ß-jellyroll (**C**). N and C notations represent the N-terminus (peptide in pink, protein in green) and C-terminus (peptide in pink) of their respective protein chains.

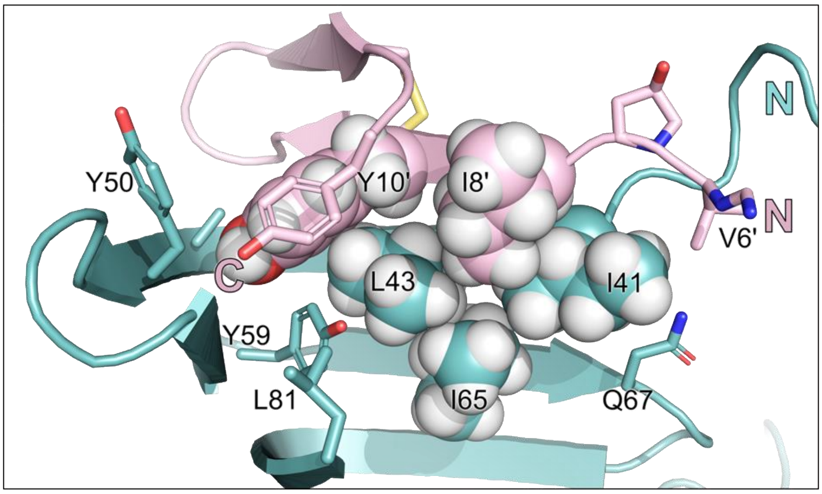

Figure S5. The intermolecular hydrophobic patch of LptAm(Ab)$_{1.0}$-**7** depicted as spheres. The described hydrophobic network consists of I8' of **7** and I41, L43 and I65 of LptAm(Ab)$_{1.0}$. Y10' is also shown in spheres for context. Important binding determinants of Enterobacteriaceae LptAm are L45 and F54, which are replaced by Y50 and Y59 in LptAm(Ab)$_{1.0}$ and are annotated in the figure. As a result, there is a marked reduction of the hydrophobic network in the ß-jellyroll core of LptAm(Ab)$_{1.0}$ compared to its Enterobacteriaceae counterparts. The N- and C-termini of **7** (pink) and LptAm(Ab)$_{1.0}$ (green) are denoted as N and C, respectively.

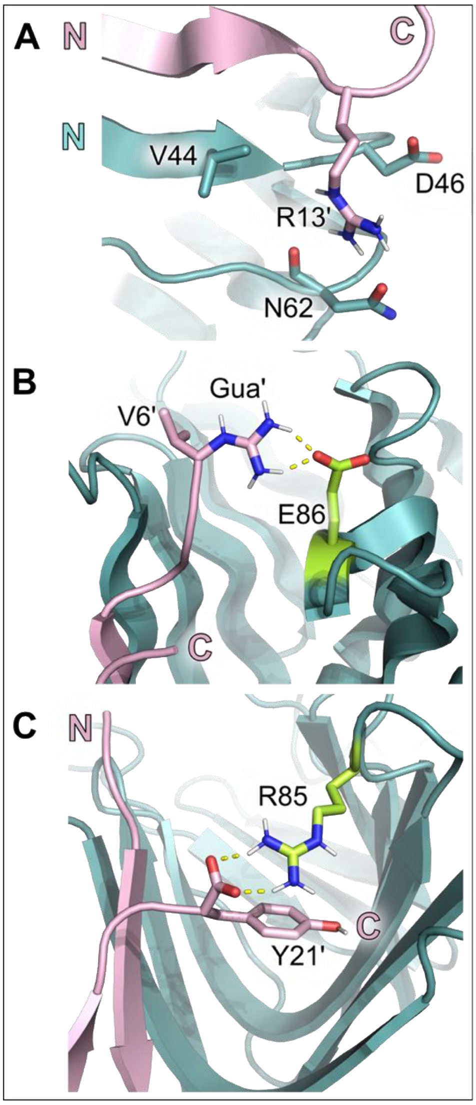

Figure S6. Postulated electrostatics between LptAm(Ab)$_{1.0}$ and **7**. R13’ (A), Gua’ (B), and Y21 (C) of **7** (pink) docks on LptAm(Ab)$_{1.0}$ (green) in a conserved manner as seen in MD simulations of *E. coli* and *K. pneumoniae* complexes. In A, the guanidine group of R13’ docks into a pocket of LptAm(Ab)$_{1.0}$ (V44, D46, N62) with intermolecular NOEs observed between V44 and R13’. Interestingly, V44 is positioned at the conserved site of Enterobacteriaceae LptAm E39 and sufficiently close to R13’ to observe NOEs. D46 and N62 of LptAm(Ab)$_{1.0}$ are analogous to D41 and N57 of Enterobacteriaceae LptAms. Similarly in B, E86 of LptAm(Ab)$_{1.0}$ was observed in some conformations to be close to the guanidine group of **7**. The same can be said for R85 and Y21’ of **7** in C. Interestingly, R85 and E86 are not in conserved positions according to the sequence alignment (Figure 6). However, due to the high flexibility observed in these residues, indicated electrostatics likely have weak or no relevance on the binding of **7** in this system. The N- and C-termini of **7** (pink) and LptAm(Ab)$_{1.0}$ (green) are denoted as N and C, respectively.

Table S6. List of all intermolecular NOEs (by type) of complex *A. baumannii* LptAm-**7**

No.	Protein side			**7**-side			NOE-type
	Res	AA	Atom	Res	AA	Atom	
1	41	ILE	H	8’	ILE	HA	bb-bb
2	43	LEU	H	10’	TYR	HA	bb-bb
3	43	LEU	H	11’	PEN	H	bb-bb
4	44	VAL	H	11’	PEN	H	bb-bb
5	45	ALA	H	11’	PEN	H	bb-bb
6	41	ILE	H	8’	ILE	HD1	bb-sc
7	43	LEU	H	11’	PEN	HG2	bb-sc
8	43	LEU	HA	11’	PEN	HG2	bb-sc
9	44	VAL	HA	11’	PEN	HG1	bb-sc
10	44	VAL	HA	11’	PEN	HG2	bb-sc
11	45	ALA	H	10’	TYR	HD	bb-sc
12	45	ALA	H	10’	TYR	HE	bb-sc
13	45	ALA	H	11’	PEN	HG1	bb-sc
14	45	ALA	H	11’	PEN	HG2	bb-sc
15	41	ILE	HB	8’	ILE	HG12	sc-sc
16	41	ILE	HD1	8’	ILE	HB	sc-sc
17	41	ILE	HG2	8’	ILE	HA	sc-sc
18	43	LEU	HB2	8’	ILE	HD1	sc-sc
19	43	LEU	HB3	8’	ILE	HG12	sc-sc
20	43	LEU	HG	10’	TYR	HD	sc-sc
21	43	LEU	HD1	8’	ILE	HG12	sc-sc
22	43	LEU	HD1	8’	ILE	HD1	sc-sc
23	43	LEU	HD2	10’	TYR	HA	sc-sc
24	43	LEU	HD2	10’	TYR	HB3	sc-sc
25	43	LEU	HD2	10’	TYR	HD	sc-sc
26	43	LEU	HD2	10’	TYR	HE	sc-sc
27	44	VAL	HB	11’	PEN	HG1	sc-sc
28	44	VAL	HB	11’	PEN	HG2	sc-sc
29	44	VAL	HG1	11’	PEN	HG2	sc-sc
30	44	VAL	HG1	13’	ARG	HA	sc-sc
31	44	VAL	HG1	13’	ARG	HG3	sc-sc
32	44	VAL	HG2	13’	ARG	HB2	sc-sc
33	45	ALA	HB	10’	TYR	HE	sc-sc
34	48	ALA	HB	10’	TYR	HD	sc-sc
35	48	ALA	HB	10’	TYR	HE	sc-sc
36	67	GLN	HE21	6’	VAN	HG1	sc-sc
37	67	GLN	HE21	6’	VAN	HG2	sc-sc
38	67	GLN	HE22	6’	VAN	HG1	sc-sc
39	81	LEU	HD1	21’	TYR	HD	sc-sc
40	81	LEU	HD1	21’	TYR	HE	sc-sc
41	81	LEU	HD2	21’	TYR	HE	sc-sc

Legend: Res, residue number; AA, amino acid; bb-bb, protein backbone – peptide backbone NOE; bb-sc, protein backbone – peptide sidechain NOE; sc – bb, protein sidechain – peptide backbone NOE; sc-sc, protein sidechain – peptide sidechain NOE.

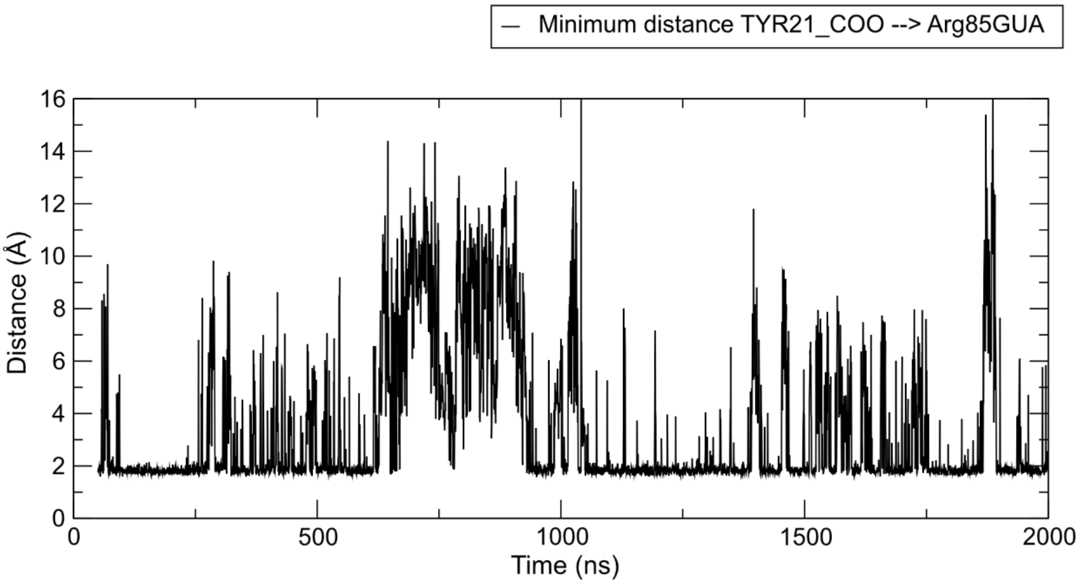

Figure S7. Minimum distances between the guanidine group of R85 of LptAm(Ab)$_{1.0}$ (Arg85GUA) and the carboxyl group of Y21’ of **7** (TYR21_COO) as a function of time during the MD simulations of complex LptAm(Ab)$_{1.0}$-**7**.

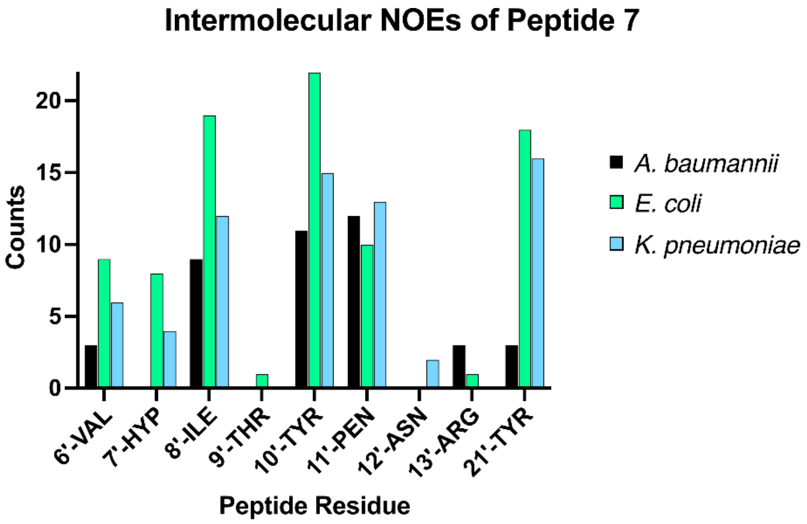

	Peptide Residue									
LptAm	6'-VAL	7'-HYP	8'-ILE	9'-THR	10'-TYR	11'-PEN	12'-ASN	13'-ARG	21'-TYR	Total
A. baumannii	3	0	9	0	11	12	0	3	3	41
E. coli	9	8	19	1	22	10	0	1	18	88
K. pneumoniae	6	4	12	0	15	13	2	0	16	68

Figure S8. Intermolecular NOEs between **7** and LptAm of *A. baumannii* (black), *E. coli* (green), and *K. pneumoniae* (blue). The table below summarizes the NOEs per peptide residue and total intermolecular NOEs observed in each system.

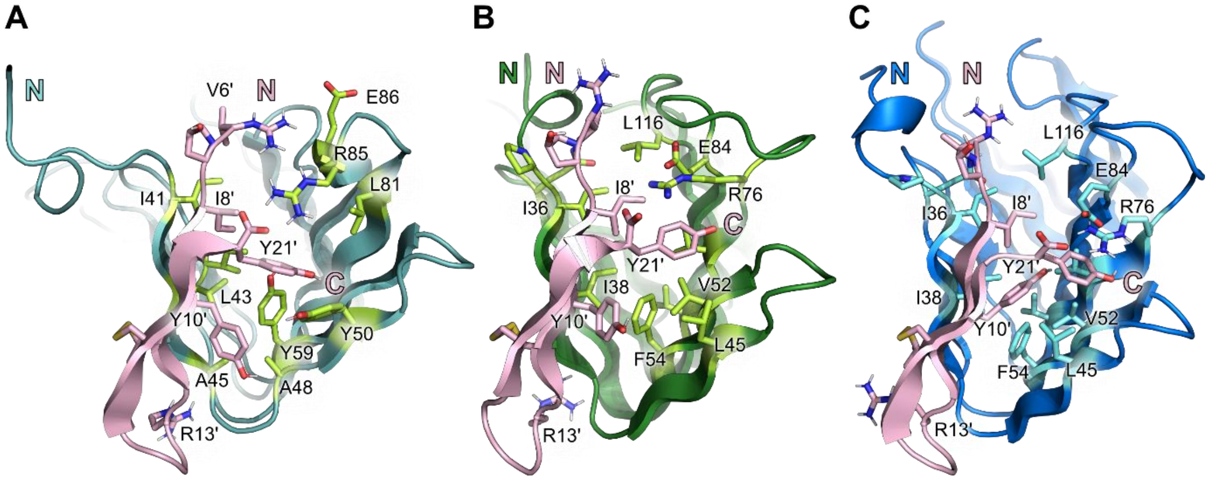

Figure S9. Comparing the binding mode of analogue **7** to LptAs of different Gram-negative pathogens. **7** binds to LptA of (A) *A. baumannii* in a similar fashion as to LptAs of Enterobacteriaceae, (B) *E. coli* and (C) *K. pneumoniae*. Side-chain residues in pink correspond to **7** whereas protein sidechains are in yellow for (A), (B) and cyan for (C).

References

1. Y. Shen, F. Delaglio, G. Cornilescu, A. Bax, TALOS+: a hybrid method for predicting protein backbone torsion angles from NMR chemical shifts. *J Biomol NMR* **2009**, *44*, 213–223.
